# 3D APT and NOE CEST-MRI of healthy volunteers and patients with non-enhancing glioma at 3 T

**DOI:** 10.1007/s10334-021-00996-z

**Published:** 2022-01-07

**Authors:** Yulun Wu, Tobias C. Wood, Fatemeh Arzanforoosh, Juan A. Hernandez-Tamames, Gareth J. Barker, Marion Smits, Esther A. H. Warnert

**Affiliations:** 1 Department of Radiology and Nuclear Medicine, Erasmus MC, Dr. Molewaterplein 40, 3015 GD Rotterdam, The Netherlands; 2 Brain Tumor Centre, Erasmus MC Cancer Institute, Rotterdam, The Netherlands; 3 Centre for Neuroimaging Science, King’s College London, London, UK

**Keywords:** CEST, APT, NOE, Non-enhancing glioma

## Abstract

**Objective:**

Clinical application of chemical exchange saturation transfer (CEST) can be performed with investigation of amide proton transfer (APT) and nuclear Overhauser enhancement (NOE) effects. Here, we investigated APT- and NOE-weighted imaging based on advanced CEST metrics to map tumor heterogeneity of non-enhancing glioma at 3 T.

**Materials and methods:**

APT- and NOE-weighted maps based on Lorentzian difference (LD) and inverse magnetization transfer ratio (MTR_REX_) were acquired with a 3D snapshot CEST acquisition at 3 T. Saturation power was investigated first by varying B_1_ (0.5–2 µT) in 5 healthy volunteers then by applying *B*_1_ of 0.5 and 1.5 µT in 10 patients with non-enhancing glioma. Tissue contrast (TC) and contrast-to-noise ratios (CNR) were calculated between glioma and normal appearing white matter (NAWM) and grey matter, in APT- and NOE-weighted images. Volume percentages of the tumor showing hypo/hyperintensity (VP_hypo/hyper,CEST_) in APT/NOE-weighted images were calculated for each patient.

**Results:**

LD APT resulting from using a *B*_1_ of 1.5 µT was found to provide significant positive TC_tumor,NAWM_ and MTR_REX_ NOE (*B*_1_ of 1.5 µT) provided significant negative TC_tumor,NAWM_ in tissue differentiation. MTR_REX_-based NOE imaging under 1.5 µT provided significantly larger VP_hypo,CEST_ than MTR_REX_ APT under 1.5 µT.

**Conclusion:**

This work showed that with a rapid CEST acquisition using a *B*_1_ saturation power of 1.5 µT and covering the whole tumor, analysis of both LD APT and MTR_REX_ NOE allows for observing tumor heterogeneity, which will be beneficial in future studies using CEST-MRI to improve imaging diagnostics for non-enhancing glioma.

## Introduction

Chemical exchange saturation transfer (CEST) imaging is a novel MRI technique with great potential for glioma diagnostics. CEST is sensitive to a reduction of bulk water signal induced by saturation transfer from exchangeable protons in a number of compounds, via which these compounds can be detected [1]. Some of these biological compounds are increased in tumor regions and can therefore potentially be used as biomarkers for tumors. One popular chosen biomarker is amide proton transfer (APT) imaging, which focuses on the amide protons of endogenous mobile proteins and peptides that resonate at 3.5 ppm [2]. Previous work has shown that APT signal is closely related to increased cell density, proliferation [3–5], and concentrations of intracellular proteins in gliosarcoma [6, 7]. Additionally, APT-weighted CEST has already been shown to be of value for clinical diagnostics in glioma, including response assessment to treatment [8–10], predicting IDH mutation status for diagnosis [5, 11], and predicting overall survival and progression-free survival [12]. However, the majority of patients studied in the current body of literature is diagnosed with high grade, enhancing gliomas (i.e., glioblastoma) [9, 13–15]. Limited studies investigate non-enhancing glioma explicitly. However, non-enhancing glioma can become quite large with molecular intratumoral heterogeneity [16] and we previously illustrated that APT-weighted CEST may play a role in imaging diagnostics in these type of tumors [17]. This is of particular importance in light of the latest World Health Organization classification for brain tumors [18], where three distinct classes of non-enhancing glioma are identified based on the identification of molecular parameters (mainly IDH mutation and 1p/19q co-deletion). These classes significantly differ in terms of prognosis and optimal treatment regime, stressing the need for optimal imaging diagnostics where APT-weighted CEST can play an important role.

To evaluate APT-weighted CEST in glioma diagnostics, early studies proposed to apply magnetization transfer ratio asymmetry (MTR_asym_) to perform APT-weighted imaging [4, 19–21]. MTR_asym_ is therefore a commonly used metric to provide CEST-weighted images by quantifying the asymmetry of Z-spectra while compensating for direct water saturation (DS) and magnetization transfer (MT) effects [1, 2, 4, 19, 20, 22, 23]. However, MT is a dominant contributor to the CEST signal when using high saturation power [24] and research is increasingly indicating that MT is not symmetric [25–27], which results in MTR_asym_ not fully correcting for MT. In addition, MTR_asym_ cannot evaluate saturation pools on opposite sides of the main resonance frequency individually, i.e., the effect of APT (3.5 ppm) and nuclear Overhauser enhancement (NOE, at − 3.5 ppm) are both reflected in MTR_asym_ at 3.5 ppm. Advanced metrics were proposed for separation of the APT and NOE signal, via multi-pool Lorentzian fitting [28] and the isolation of individual CEST effects by the Lorentzian difference (LD) [17, 28–33]. In addition, relaxation-compensated inverse magnetization transfer ratio (MTR_Rex_ [34, 35]) combined with Lorentzian fitting was proposed in glioma imaging at clinical field strength (3 T) to account for spillover effects that cannot be compensated by LD analysis [13, 28, 30, 36–38].

The NOE effect arises from through-space inter- and intramolecular dipole–dipole magnetization transfer between the water protons and aliphatic and olefinic components of mobile proteins, peptides, metabolites, and lipids, which is different than conventional MT contrast [1]. NOE imaging has also shown potential to serve as a novel imaging biomarker for glioma grading [39], predicting early progression after treatment [8, 9], and mapping tumor heterogeneity [40]. Moreover, Paech et al. showed that NOE-weighted CEST contrast also correlates with histopathological assessments of cell density in glioblastoma [41].

Acquisition of CEST metrics via a Lorentzian fitting approach requires densely sampled frequency offsets across a wide range, which inherently increases scan time and hampers application in clinical settings. Recently, a snapshot 3D readout has been developed to obtain CEST contrast for an image volume covering several slices with an acquisition time of 7 s per irradiation frequency offset [42, 43]. This efficient acquisition allows dense sampling of Z-spectra with a wide range within clinically feasible scan time. When considering clinical feasibility of CEST in non-enhancing glioma imaging, it is important to understand the application of LD-based APT and NOE studies under an appropriate saturation module at 3 T. Our initial work showed that APT-weighted signal isolated from Lorentzian fitting of DS has the ability to map tumor heterogeneity by identifying areas of hyperintensity within non-enhancing glioma, even with a suboptimal saturation and acquisition scheme [17]. Building on this work, we now use 3D snapshot CEST [42, 43] and investigate APT and NOE imaging in non-enhancing glioma based on Lorentzian fitting of DS and MT. This was done first in healthy volunteers to fine tune the saturation module of the CEST acquisition scheme. Subsequently, we applied two different saturation powers (*B*_1_) and applied LD and MTR_REX_ analysis in 10 patients diagnosed with non-enhancing gliomas to investigate and compare the ability of APT- and NOE-weighted CEST-MRI to map tumor heterogeneity.

## Materials and methods

Our studies were conducted in compliance with the Declaration of Helsinki and under approval of the institutional ethics committee of the Erasmus MC (Rotterdam, NL), which is 1 out of 18 accredited medical research ethics committees in the Netherlands. For the healthy volunteer study, we recruited five subjects (male/female = 1/4, mean age: 24.9 years). For the patient study, ten subjects with non-enhancing glioma were recruited. Patients were recruited as part of the Imaging Genomics study [44] and were scanned at maximum 2 days before surgical resection, where biopsies were taken for tumor stratification. Patient characteristics can be seen in Table 1.

**Table 1:** Information of patients included in our study

Subject	Age	M/F	Grade	Type	1p/19q co-deletion	IDH mutation
1	32	M	2	Oligodendroglioma	True	True
2	28	F	2	Astrocytoma	False	True
3	31	M	2	Astrocytoma	False	True
4	24	M	3	Oligodendroglioma	True	True
5	35	M	2	Astrocytoma	False	True
6	37	M	2	Astrocytoma	False	True
7	30	F	2	Astrocytoma	False	True
8	52	M	4	Glioblastoma	False	False
9	46	F	2	Oligodendroglioma	True	True
10	54	M	3	Oligodendroglioma	True	True

### Image acquisition

All measurements were performed on a 3 Tesla scanner (Discovery750, General Electric, Chicago, USA) with a 32-channel head coil. A 3D snapshot CEST sequence [43] was used with the following acquisition parameters: TR = 7 ms, TE = 3.2 ms, field of view = 220 × 180 × 42 mm^3^ and matrix size 128 × 104 × 14 for a resolution of 1.7 × 1.7 × 3 mm^3^, acceleration factor of 4, and flip angle 6 degrees. Pulse-train saturation consisted of a train of 80 Gaussian-shaped radiofrequency (RF) pulses, each pulse with pulse time 20 ms and interpulse delay 20 ms (50% duty cycle), resulting in 3.2 s total saturation time.

Z-spectra were obtained with 53 frequency offsets: at ± 100 ppm, from ± 50 to ± 20 ppm in steps of 10 ppm, from ± 10 to ± 5 ppm in steps of 1 ppm, from ± 4 to ± 1 in steps of 0.5 ppm, and from ± 0.5 to 0 ppm in steps of 0.25 ppm. In addition, two images were obtained with saturation pulses at − 300 ppm (i.e., at a frequency at which any saturation effects are expected to be negligible). The first was discarded as the signal had not reached equilibrium. The second image was used to normalize the Z-spectrum. The CEST scan took 4 min and 40 s.

To investigate appropriate *B*_1_ saturation power of the CEST sequence, we applied varying *B*_1_ saturation power (0.5, 0.75, 1, 1.5, 2 µT) in the scan of each healthy volunteer. Additionally, we investigated B_1_ saturation duration and acquired two more CEST acquisitions using 40 or 60 RF pulses under the *B*_1_ of 2 µT. In patients diagnosed with glioma, we applied two *B*_1_ saturation powers (0.5, 1.5 µT) and 80 RF pulses to investigate tumor imaging and tumor-white matter separation dependent on *B*_1_. Note that the saturation powers used are stated as the root mean square *B*_1_ (RMS *B*_1_) across the saturation train.

### Data analysis

Motion correction of the CEST image series was done by linear registration of each image within a series to the 6 ppm image (mcflirt [45], within the free online software FMRIB Software Library (FSL) v5.0.9 [46]). After motion correction, the CEST image at 6 ppm was linearly registered to the *T*_1_-weighted post-contrast image, resulting in transformation matrices from CEST to *T*_1_ post-contrast space. These matrices were inverted and used to transform the regions of interest (ROI) based on structural images into the CEST space. All alignments were linear registrations performed with FLIRT [45] within FSL.

In-house written scripts in Matlab (Version R2015a, the Mathworks, Inc., Natick, MA) were used to perform voxel-wise analysis for the CEST image series. Z-spectra were calculated by dividing the images acquired with off-resonance saturation pulses by the *S*_0_ image (Eq. 1). 2-D adaptive noise-removal filtering based on [47] was applied to remove the noise of Z-spectra

1 $$Z\left(\Delta \omega \right)=\frac{{S}_{\mathrm{sat}}\left(\Delta \omega \right)}{{S}_{0}}.$$

A Lorentzian fitting scheme similar to [48] was applied in our study. The fitting based on a two-pool model (Eq. 2) was performed to define reference Z-spectra by fitting DS and MT effects to the Z-spectra

2 $$Z_{{{\text{ref}}}} \left( {\Delta \omega } \right) = 1 - A_{{{\text{water}}}} \frac{{L_{{{\text{water}}}}^{2} /4}}{{L_{{{\text{water}}}}^{2} /4 + \left( {\Delta \omega - \delta _{{{\text{water}}}} } \right)^{2} }} - A_{{{\text{MT}}}} \frac{{L_{{{\text{MT}}}}^{2} /4}}{{L_{{{\text{MT}}}}^{2} /4 + \left( {\Delta \omega - \delta _{{{\text{MT}}}} } \right)^{2} }} + b.$$

Here, $A$ is the Lorentzian amplitude, $L$ is the Lorentzian width, $\delta$ is the center frequency, and $b$ is the total shift. Only frequency offsets considered to be affected by the background signal (from DS and MT effects) were used for fitting. For the fit of DS, the images acquired at ± 1, ± 0.5, ± 0.25, and 0 ppm were used. For fitting the broad MT effect, the images acquired at ± 100, ± 50, ± 40, ± 30, ± 20, ± 10, ± 9.5, ± 9, ± 8.5 ppm were used.

LD analysis was used to determine CEST effects: the fitted DS and MT effects were subtracted from the full Z-spectra. The two-pool Lorentzian fit was also used for B0 inhomogeneity correction. In each voxel, LD and Z-spectra were shifted by the frequency shift of the minimum value of the Lorentzian fit. Then, both of them were calculated by interpolating the value from − 100 to 100 ppm to a resolution of 0.1 ppm and, for each frequency shift Δ*ω*, averaging LD between Δ*ω* − 0.2 ppm and Δ*ω* + 0.2 ppm. As a result, *B*_0_-corrected LD was obtained, from which the APT (3.5 ppm) and NOE (− 3.5 ppm) weighted LD maps were generated. After LD analysis, *Z*_ref_ and *Z* were used to calculate the MTR_REX_ [34, 35, 48] where APT and NOE signal at + 3.5 and − 3.5 ppm

3 $$\mathrm{LD}={Z}_{\mathrm{ref}}-Z,$$

4 $${\mathrm{MTR}}_{\mathrm{REX}}=\frac{1}{Z}-\frac{1}{{Z}_{\mathrm{ref}}}.$$

Here, *Z*_ref_ was calculated from two-pool fitting as a reference signal, and *Z* was the normalized *B*_0_-corrected Z-spectra. In the following text, when we talked about APT/NOE imaging under a certain CEST metrics, we refer LD APT/NOE or MTR_REX_ APT/NOE to the APT/NOE-weighted CEST imaging based on LD or MTR_REX_, respectively.

### Region of interest analysis

In the healthy volunteers, whole brain white matter (WM) and grey matter (GM) were used as ROI. In the patients, the contralateral normal appearing WM (NAWM) and GM (NAGM), and tumor were used as ROI. WM/GM tissue segmentation was performed automatically by FAST [49] based on brain extracted *T*_1_-weighted structural images. The whole brain masks were manually divided into hemispheres. For tumor segmentation in patients, we aligned pre-contrast *T*_1_-weighted, *T*_2_-weighted, and FLAIR images into the space of post-contrast T_1_-weighted image using the Elastix toolbox (version 2.5) [50]. Based on these four structural images, the tumor masks for non-enhancing glioma were automatically delineated using HD-GLIO [51, 52]. All tumor segmentations were checked by an experienced neuroradiologist. The WM, GM, and tumor ROI were then aligned into CEST space. We computed the mean value of LD APT/NOE and MTR_REX_ APT/NOE for each ROI in each subject. To evaluate the tissue separation of CEST imaging between ROI (tumor and NAWM/GM), we computed tissue contrast (TC) and contrast-to-noise ratio (CNR) for each patient based on Eqs. 3 and 4 based on LD

5 $${\mathrm{TC}}_{ \mathrm{tumor},\mathrm{NAWM}/\mathrm{GM}}=\mathrm{mean} {\mathrm{LD}}_{\mathrm{tumor}}-\mathrm{mean} {\mathrm{LD}}_{{\mathrm{NAWM}}/{\mathrm{GM}}},$$

6 $${\mathrm{CNR}}_{\mathrm{tumor},\mathrm{NAWM}/\mathrm{GM}}=\frac{{\mathrm{TC}}_{\mathrm{tumor},\mathrm{NAWM}/\mathrm{GM}}}{\sqrt{{\mathrm{std}}_{\mathrm{tumor}}^{2}+{\mathrm{std}}_{\mathrm{NAWM}/\mathrm{GM}}^{2}}}.$$

To evaluate the extent of hyper/hypointensity of LD APT/NOE and MTR_REX_ APT/NOE within the tumor per patient, a patient-specific threshold was determined as reported in [17]

7 $${\mathrm{LD}}_{\mathrm{thresh},\mathrm{hyper}}>\overline{{\mathrm{LD} }_{\mathrm{APT},\mathrm{ NAWM}}}+2\times {\mathrm{std}}_{\mathrm{APT},\mathrm{ NAWM}},$$

8 $${\mathrm{LD}}_{\mathrm{thresh},\mathrm{hypo}}<\overline{{\mathrm{LD} }_{\mathrm{APT},\mathrm{ NAWM}}}-2\times {\mathrm{std}}_{\mathrm{APT},\mathrm{ NAWM}},$$

where $\overline{{\mathrm{LD} }_{\mathrm{APT},\mathrm{ NAWM}}}$ is the average LD APT in NAWM. The percentage of hyper/hypointense voxels (VP_hyper/hypo,CEST_) was calculated by dividing the numbers of voxels below ${\mathrm{LD}}_{\mathrm{thresh},\mathrm{hyper}/\mathrm{hypo}}$ within the tumor region by the total number of voxels covering the tumor region which calculated from the structural images for each patient. To assess TC, CNR, and VP_hyper/hypo,CEST_ for MTR_REX_, Eqs. 5–8 were calculated by substituting LD for MTR_REX_.

### Statistical analysis

All statistical analyses were performed by SPSS (IBM Corp. Released 2017. IBM SPSS Statistics for Windows, Version 25.0. Armonk, NY: IBM Corp). For comparison of each *B*_1_ group in the healthy volunteers study, one-way ANOVA was applied to investigate the effects of different *B*_1_ powers on mean LD APT/NOE in each ROI. Post-hoc analysis was applied to compare the differences of group mean values between *B*_1_ groups.

In the patient study, mean TC and mean CNR_tumor,NAWM/NAGM_ based on LD APT/NOE and MTR_REX_ APT/NOE were calculated for all subjects under each *B*_1_ power. Paired *T* tests were used to compare mean TC_tumor,NAWM/NAGM_ and CNR_tumor,NAWM/NAGM_ under different B_1_ powers in patients. One-sample *T* tests were performed for each TC and CNR. Paired *T* tests were used to compare VP_hyper/hypo,CEST_ under different CEST metrics in patients. One-sample *T* tests were performed for each VP_hyper/hypo,CEST_ for LD APT/NOE and MTR_REX_ APT/NOE. In all statistical analyses, *p* < 0.05 was considered a significant difference.

## Results

### Healthy volunteers study

**Fig. 1:**
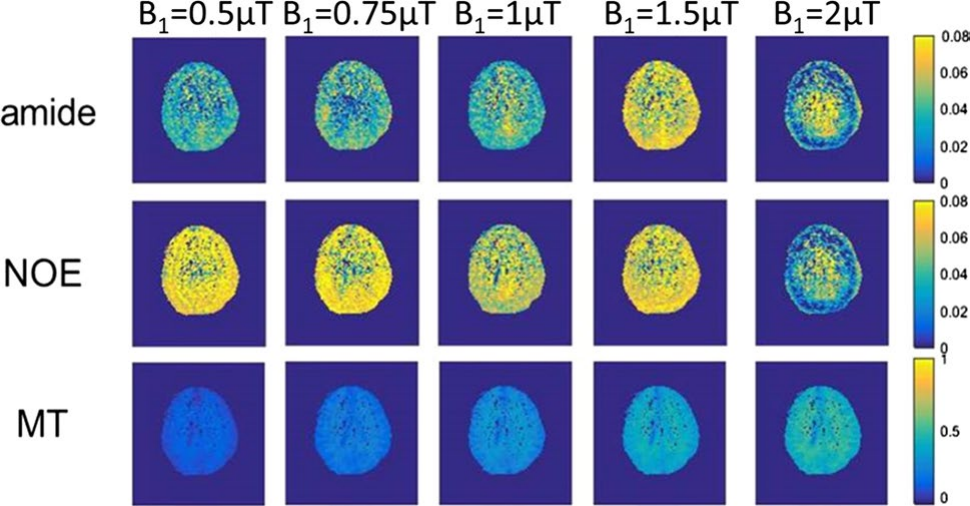
Example map of different effects, from a single brain slice in a healthy volunteer under different *B*_1_ powers. Each row corresponding to an effect (from top to bottom: LD APT at 3.5 ppm, LD NOE at − 3.5 ppm, amplitude of MT pool). Each column corresponds to a *B*_1_ power

**Fig. 2:**
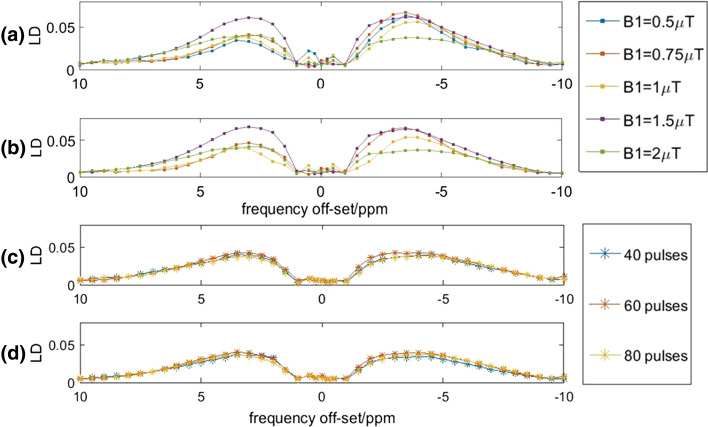
Influence of *B*_1_ power (**a**, **b**) and number of RF pulses on LD (**c**, **d**, with *B*_1_ = 2 µT) spectrum. The LD was averaged across the WM (**a**, **c**) and GM (**b**, **c**) in the brain of a single healthy volunteer

Examples of LD APT, LD NOE, and MT maps acquired with different *B*_1_ power are shown in Fig. 1. The mean LD spectra for WM and GM of one volunteer and with different *B*_1_ powers and RF pulses are shown in Fig. 2. These results illustrate that LD APT and NOE were both stronger when using 1.5 µT compared to using 2 µT. The number of RF pulses used made only a limited difference to the Z-spectra.

**Fig. 3:**
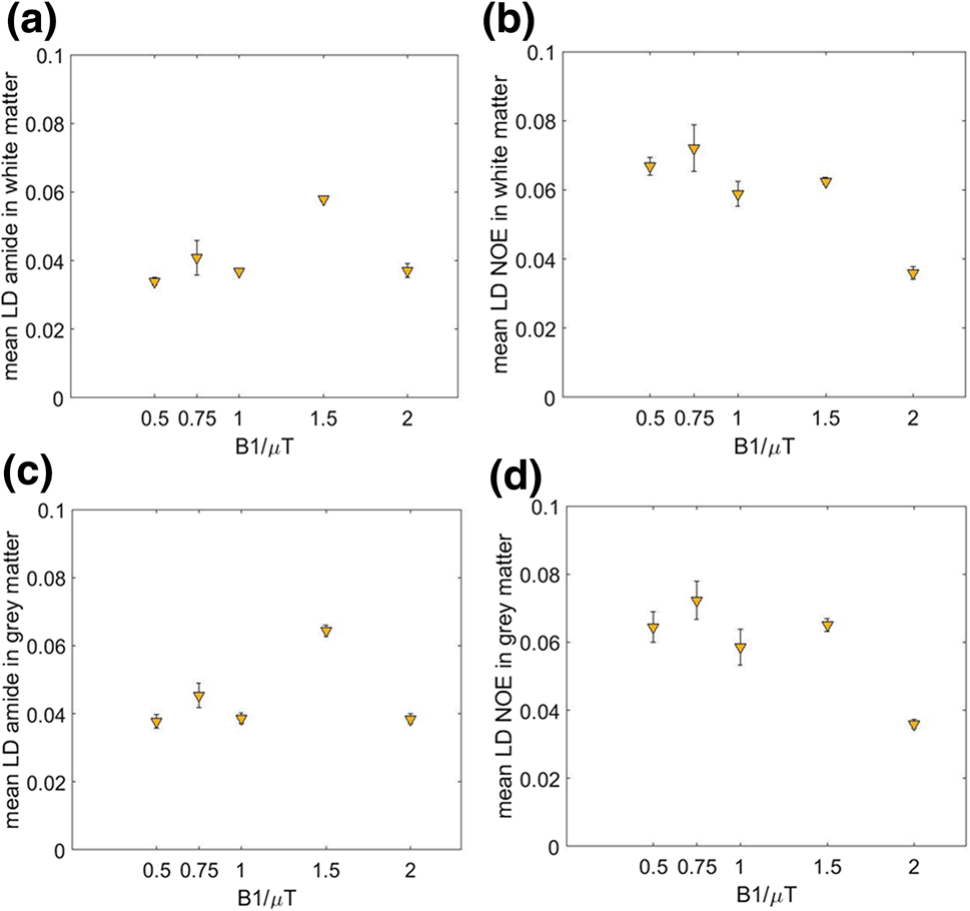
Group averaged LD APT (**a**, **c**) and NOE (**b**, **d**) in WM (top row) and GM (bottom row). Error bars represent standard deviation. LD APT was significantly higher (*p* < 0.05) in WM and GM at 1.5 µT compared to any other B_1_ used. LD NOE was significantly lower (*p* < 0.05) in WM at 2 µT compared to any other *B*_1_ used

Group averaged LD APT/NOE in WM and GM per B_1_ power used are shown in Fig. 3. The one-way ANOVA showed a significant effect of *B*_1_ power on LD APT/NOE in WM and GM (*p* < 0.05). In post hoc analysis, it showed that LD APT was significantly higher (*p* < 0.05) in WM and GM at 1.5 µT compared to any other *B*_1_ used. LD NOE was significantly lower for 2.0 µT in WM compared to any other *B*_1_ power used (*p* < 0.05). The same trend for lowest LD NOE at 2 µT was observed in GM, but this finding was not significant.

### Patient study

**Fig. 4:**
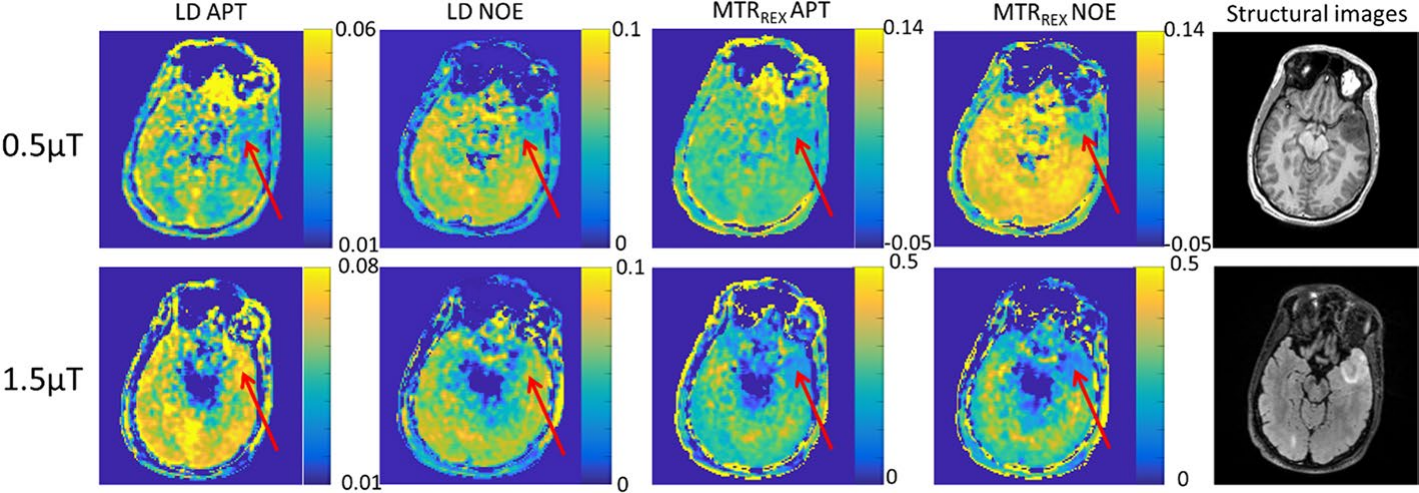
Example brain slice of patient 3 (first row: LD APT, LD NOE, MTR_REX_ APT, MTR_REX_ NOE, under 0.5 µT and T_1_ pre-contrast image; second row: LD APT, LD NOE, MTR_REX_ APT, MTR_REX_ NOE under 1.5 µT and FLAIR image

**Fig. 5:**
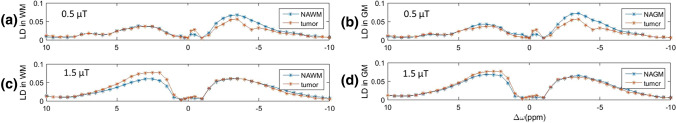
Influence of B_1_ power on mean LD in NAWM (**a**, **c**) or NAGM (**b**, **d**) compared with mean LD in tumor in the brain of a single subject. **a**, **b**: *B*_1_ = 0.5 µT, **c**, **d**: *B*_1_ = 1.5 µT

Examples of resulting images from one patient are shown in Fig. 4. It can be seen that in the tumor region, LD APT is hyperintense under 1.5 µT, while LD NOE is hypointense under 0.5 µT. For MTR_REX_, APT and NOE hypointensity in the tumor region was found, which was stronger when using a B_1_ of 1.5 µT compared to 0.5 µT. The relation between LD APT/NOE and B_1_ saturation power was different (Fig. 5). The mean LD spectra of the tumor and NAWM for one patient are plotted in Fig. 5. With increasing B_1_, the increase in LD downfield from on resonance (ppm > 0) was larger in the tumor than in NAWM/NAGM, but the LD upfield from on resonance (ppm < 0) showed a stronger decrease in NAWM/NAGM than in the tumor (comparing top and bottom rows in Fig. 5).

**Fig. 6:**
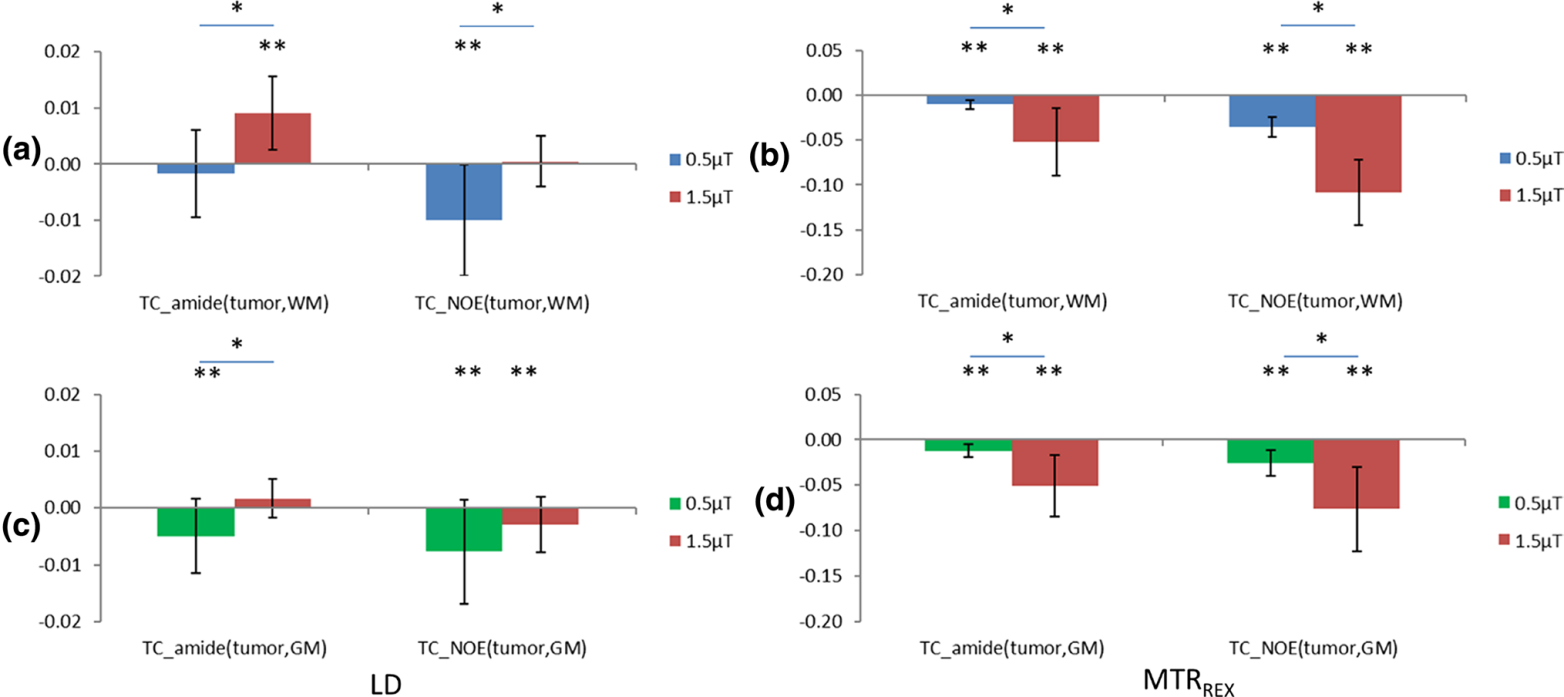
Comparison of group averaged TC_tumor,NAWM_ (**a**, **b**) and TC_tumor,NAGM_ (**c**, **d**) for LD APT/NOE (left column) and for MTR_REX_ APT/NOE(right column) each for the two *B*_1_ powers used (in color). Error bars represent standard deviations. *Significantly different between *B*_1_ powers, *p* < 0.05, **significantly different from 0, *p* < 0.05

**Fig. 7:**
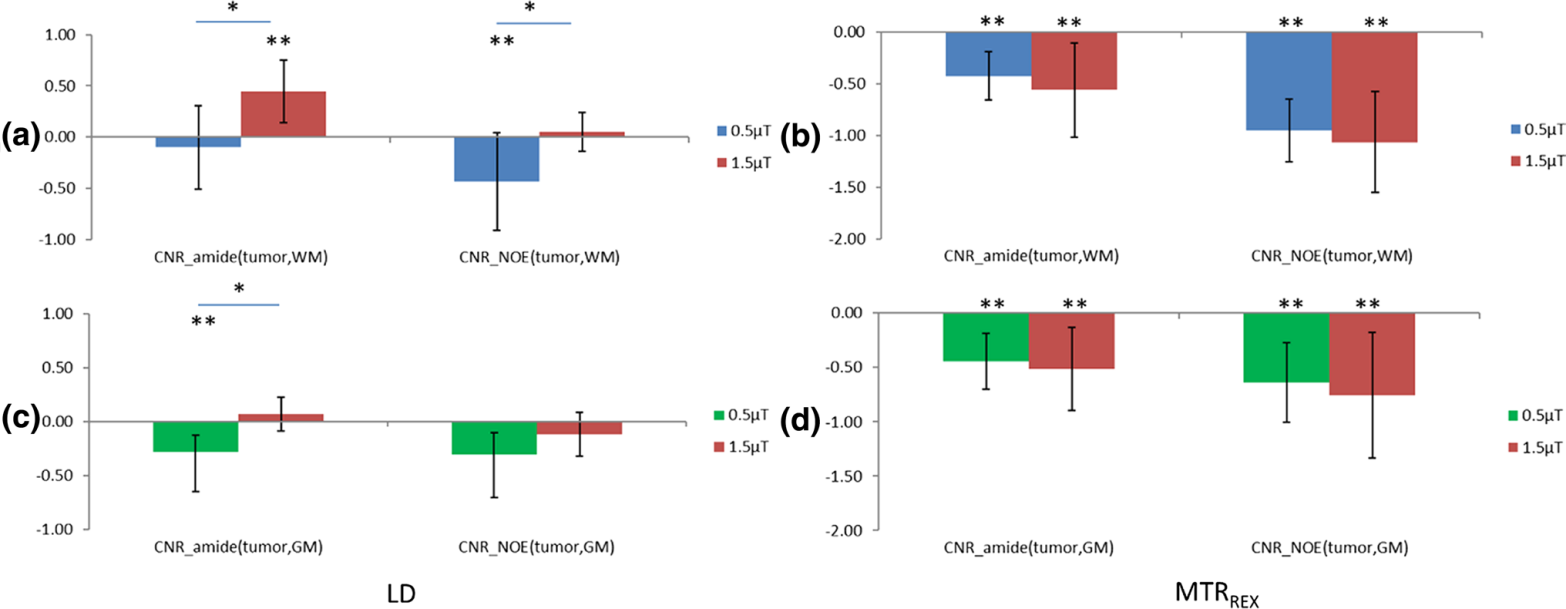
Comparison of group averaged CNR_tumor,NAWM_ (**a**, **b**) and CNR_tumor,NAGM_ (**c**, **d**) for LD APT/NOE (left column) and for MTR_REX_ (right column), each for both B_1_ powers used. Error bars represent standard deviations. *Significantly different between *B*_1_ powers, *p* < 0.05, **significantly different from 0, *p* < 0.05

To investigate whether tissue contrasts between the tumor and NAWM/NAGM were significant for APT- and NOE-weighted imaging separately, one-sample *t* tests were applied and the results are shown in Figs. 6 and 7. LD APT under 1.5 µT provided significantly positive TC_tumor,NAWM_ and CNR_tumor,NAWM_, while under 0.5 µT, it provided significantly negative TC_tumor,NAGM_ and CNR_tumor,NAGM_ (Figs. 6 and 7, left side, *p* < 0.05, *N* = 10). LD NOE under 0.5 µT provided significantly negative TC_tumor,NAWM_ and CNR_tumor,NAWM_, and also significantly negative TC_tumor,NAGM_ (Figs. 6 a, c and 7a, right side, *p* < 0.05, *N* = 10). Under 1.5 µT, it provided significantly negative TC_tumor,NAGM_ (Fig. 6c, right side, *p* < 0.05, *N* = 10). MTR_REX_ APT/NOE provided significant negative TC_tumor,NAWM/NAGM_ and CNR_tumor,NAWM/NAGM_ under 0.5 and 1.5 µT. MTR_REX_ showed a stronger increase in both TC and CNR compared with LD (Fig. 6/7, a, c versus b, d).

To compare LD APT/NOE between the two different *B*_1_ powers, paired *t* tests were applied and the results can be seen in Figs. 6 and 7. For LD APT, TC_tumor,NAWM_ was positive for *B*_1_ = 1.5 µT and significantly higher than for 0.5 µT (Fig. 6a, left side, *p* < 0.05, paired *t* test, *N* = 10). For LD NOE, TC_tumor,NAWM_ was negative for *B*_1_ = 0.5 µT and significantly lower than for 1.5 µT (Fig. 6a, right side, *p* < 0.05, paired *t* test, *N* = 10). In GM, TC_tumor,NAGM_ of LD NOE in 0.5 µT was lower than 1.5 µT, both are negative. In comparing CNR_tumor,NAWM/NAGM_ between the two B_1_ powers, the same results were found which are shown in Fig. 7. For MTR_REX_ APT/NOE, TC_tumor,NAWM/NAGM_ was negative and significantly lower at 1.5 µT than 0.5 µT (Fig. 6b, d, *p* < 0.05, paired *t* test, *N* = 10). In comparing CNR_tumor,NAWM/NAGM_ of MTR_REX_, no significant difference was found between 0.5 and 1.5 µT.

**Fig. 8:**
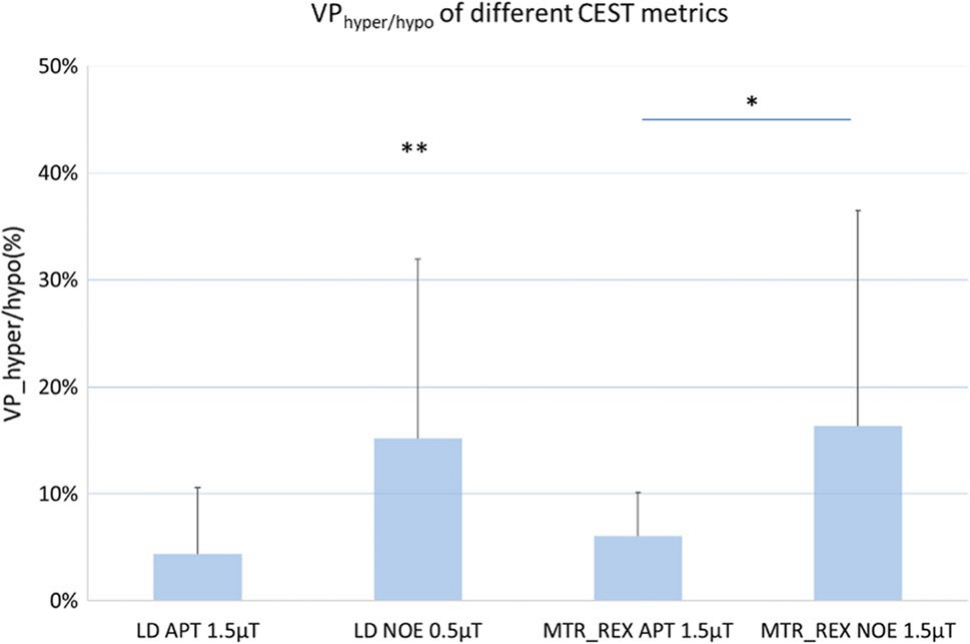
Group averaged VP_hyper/hypo_ for LD APT/NOE and for MTR_REX_ APT/NOE. Error bars represent standard deviations. *Significantly different between MTR_REX_ APT and NOE imaging, *p* < 0.05, **significantly different from 0, *p* < 0.05

In the group analysis of VP_hyper/hypo,CEST_ (Fig. 8), LD NOE showed significant VP_hypo,CEST_ (15.1% ± 16.7%, tested with one-sample *t* tests, *p* < 0.05, *N* = 10). MTR_REX_ NOE showed significant higher VP_hypo,CEST_ than MTR_REX_ APT (16.3% ± 23.7% and 6.0% ± 13.4%, respectively, tested with paired *t* test, *p* < 0.05, *N* = 10). No significant difference was found in VP_hypo,CEST_ between LD NOE and MTR_REX_ NOE. No significant difference was found in the contrast of LD APT (with VP_hyper,CEST_ of 4.3% ± 6.2%) and MTR_REX_ APT. No significant difference was found comparing VP_hyper,CEST_ resulting from LD APT vs LD NOE.

## Discussion

We found that LD APT with using a *B*_1_ saturation power of 1.5 µT provided significant positive TC_tumor,NAWM_ and MTR_REX_ NOE with using a *B*_1_ of 1.5 µT provided significant negative TC_tumor,NAWM_. We also found that MTR_REX_ NOE with using a *B*_1_ of 1.5 µT resulted in 16% of the tumor volume to show hypointense signal, which was significantly larger than for MTR_REX_ APT. Here, we improved upon our previous work investigating the use of CEST in non-enhancing glioma [17]. The application of a snapshot sequence [43] introduced an even faster acquisition, which allowed us to increase the range of frequency offsets acquired into − 100 to 100 ppm. This enabled improved MT fitting via the application of a two-pool Lorentzian fitting approach for estimation of LD APT/NOE and MTR_REX_ APT/NOE. Moreover, we applied lower B_1_ saturation powers than the one we used previously [17] to increase the weighting of APT and NOE by decreasing the DS and MT effects.

### Healthy volunteer study

Using a *B*_1_ power of 1.5 µT showed strongest contrast for LD APT, both in WM and GM. The reason for the increased trend for LD APT from 0.5 to 1.5 µT is likely that higher saturation powers enhance the CEST effect by increasing saturation efficiency *α* [53, 54]. However, there is a trade-off for CEST imaging to reach a high APT/NOE effect. Contribution from MT to the saturated CEST signal becomes larger than APT and NOE effects under high B_1_ power, as suggested in previous simulation work [24, 33]. Our data indicate that this might occur after reaching *B*_1_ power of 2 µT and that the contribution of MT in WM is approximately ten times as high as APT/NOE effects. This increases the difficulty of isolating APT/NOE signal, hampering accurate fitting of APT/NOE.

For NOE imaging, we found the highest contrast for using *B*_1_ saturation powers smaller than 2 µT, with the maximum for 0.5 µT which is in line with results from Deshmane et al. [43]. Based on the group averaged LD in Fig. 3, we considered an appropriate *B*_1_ power for imaging LD APT between 1 and 2 µT and a *B*_1_ of < 1 µT for NOE. We selected 0.5 and 1.5 µT for the subsequent patient study. We found no differences in LD APT/NOE when reducing the number of RF pulses from 80 to 40. This can be explained by already having sufficient saturation when using 40 RF pulses in healthy tissue, which helps saving 1.6 s per CEST image. However, we kept using 80 pulses for glioma imaging in the following patient study to ensure sufficient signal to noise, accepting the slightly longer acquisition time. In addition, in our current set-up we found that using a B_1_ saturation power of 2 µT led to CEST contrast maps that are visibly affected by B_1_ inhomogeneity. This partly explains why in our set-up using this particular B_1_ power was suboptimal and hence we did not use this saturation power when assessing CEST contrasts in patients.

### Patient study

Our relatively low LD APT values for glioma are in line with what is expected based on previous studies. Note that here we included patients diagnosed with non-enhancing tumors of which nine out of ten were IDH mutated. Finding limited increases in LD APT is therefore in line with previous studies finding isointense or moderate increases in APT-weighted signal in non-enhancing or low-grade glioma [17, 55, 56]. We can expect higher APT-weighted signal in high-grade gliomas [3, 55], and thus, better TC_tumor,NAWM_ and CNR_tumor,NAWM_ will be found in datasets including high-grade gliomas, which often show enhancement on T_1_-weighted post-contrast images. In addition, care should be taken in comparing the APT-weighted values across different literature. One of the reasons is the definition of *B*_1_ power. In our study, we used RMS *B*_1_ across the Gaussian-shaped saturation train, similar as used by some other studies [57, 58]. However, it can also be defined by amplitude of *B*_1_ pulses used [59] or the mean across the *B*_1_ pulse train [43]. Another reason for finding discrepancies within the literature is the use of different metrics to evaluate APT-weighted signal, such as MTR_asym_, that are sensitive to other sources than LD APT analysis. This can lead to a different evaluation of APT-weighted signal and different effect sizes to differentiate tumor tissue from NAWM, as investigated previously [17].

In tumor, LD APT showed a stronger increase than LD NOE with increasing B1 from 0.5 to 1.5 µT. This different sensitivity to B_1_ illustrates the need of isolating CEST effects for APT and NOE. It also causes a lower TC based on LD NOE at 1.5 µT than 0.5 µT. It is hypothesized that there are more proteins present for proton exchange in the tumor region than in NAWM, which is likely the main contributor for hyperintense APT signal observed in LD APT at 1.5 µT in our study [4, 7, 60, 61]. A larger increase of LD APT was observed within the tumor region than the NAWM when increasing *B*_1_ power. This can be the reason of the larger LD-based positive TC_tumor,NAWM_ (~ 1%) under 1.5 µT. The saturation time we used was 3.2 s and sufficient to reach steady state, which can be seen from the LD value near 0 at far-off frequency offsets, achieving the condition for allowing MTR_REX_ analysis [36]. MTR_REX_ APT showed higher TC and CNR than LD, both under 0.5 and 1.5 µT, which is most likely due to the correction of spillover effects in MTR_REX_ analysis. Interestingly, we found hypointense MTR_REX_ APT in the tumor region with using a *B*_1_ of 1.5 µT, while for LD APT with the same *B*_1_ power, we found hyperintense regions within the tumor. This is in line with previous research at 7 T, where LD APT provided significant positive TC_tumor,NAWM_ and was decreased with spillover correction [30]. Moreover, the authors explained that APT and NOE imaging in patients with newly diagnose glioblastoma might be showing opposite behavior in tumor contrast because of increased small mobile proteins and decreased large proteins [13]. In our study on non-enhancing glioma, this hypothesis may be reflected by positive TC_tumor,NAWM/NAGM_ of LD APT and negative TC_tumor,NAGM_ of LD NOE at 1.5 µT, but not by our MTR_REX_ results. Future validation of LD and MTR_REX_ in non-enhancing glioma, for instance by increasing the sample size, is needed to investigate whether our current findings will hold.

The LD NOE map showed increases in NAWM and NAGM at 0.5 µT compared to 1.5 µT. A previous study including a low-power saturation experiment (*B*_1_ = 0.6 μT) at 3 T showed that NOE signal is significantly lower in the core of a brain tumor compared to contralateral white matter [43]. With using a B_1_ power of 1.5 µT, we found hypointensity in the tumor area and larger negative TC_tumor,NAWM_ for MTR_REX_ NOE than for LD NOE, which is again likely reflecting a stronger correction for the spillover effect by MTR_REX_ analysis. Such high MTR_REX_-based TC_tumor,NAWM/NAGM_ is in agreement with previous work using MTR_REX_ NOE at 3 T [13, 36]. The origin of hypointense NOE signal can come from low concentration of lipids [30] or the presence of semi-solid proteins with lower mobility in the tumor region. Another origin of such decreased NOE signal can be unfolding of proteins in necrotic regions of the tumor [62]. However, increased numbers of misfolded proteins are often seen in cells with high proliferation rate found in gadolinium enhancing areas, which are of course not present in our non-enhancing glioma data set.

Despite the larger effect size of tissue separation by MTR_REX_, similar VP_hyper/hypo,CEST_ was found when comparing between LD APT and MTR_REX_ APT at 1.5 μT. This suggests that LD APT analysis still maintains the ability to detect the possible most aggressive region of tumor, despite fully correcting for the spillover effect. Note that the hyper- and hypointense regions resulting from both MTR_REX_ and LD confirm the ability of CEST imaging to assess intratumoral heterogeneity of non-enhancing glioma at 3 T. Compared with APT-weighted imaging, higher VP_hypo,CEST_ from NOE-weighted imaging may arise from mixed contributions from lipids, low-mobile proteins, and protein unfolding, which can provide additional information for the prediction of physiological environment of the tumor region. With the application of MTR_REX_, one CEST scan could be sufficient to include both APT and NOE imaging with a clinical feasible time (< 5 min). The clinical potential of assessing CEST metrics across a tumor volume will lie in assessing which area in the tumor is more aggressive/reflective of the most aggressive part, which can aid taking a biopsy for most accurate diagnosis. In future, this may even aid in identifying tumor regions that already have microscopic tumor invasion, beyond the tumor ROI currently identified solely on structural scans. Further research is needed confirming validation of APT/NOE CEST with targeted biopsies that are not only analyzed by histopathological markers of cell density and proliferation, but also for the underlying protein content with proteomics in human glioma. In combination with reproducibility measurements and prospective, multicenter trials for CEST-based biomarkers such research will further stimulate clinical applications of CEST-MRI, including the use of APT/NOE-weighted CEST for diagnosis and assessment and prediction of therapy response.

A limitation of this study is the small sample size of ten patients, which inhibits investigations of using LD/MTR_REX_ APT and NOE for diagnosis of non-enhancing glioma. Additionally, we used two-pool Lorentzian fitting and MTR_REX_ to evaluate APT/NOE CEST effects. There are other advanced CEST metrics, for example apparent exchange-dependent relaxation (AREX), that compensate for changes in *T*_1_ relaxation times. However, application of such metrics needs to include *T*_1_ mapping in the scan protocol, which requires additional sequences and longer acquisition. Suggested by Goerke et al. 36, with the prior knowledge about large spectral range of CEST effects (> ± 6 ppm), one can perform Lorentzian fitting at 3 T without additional advanced fitting approaches such as multistep fitting or MT fitting with special line shapes [43, 63–65]. Finally, here, we did not apply a multi-pool fitting approach (> 2 pools) due to the mixed and broadened CEST effects at 3 T. Thus, our LD/MTR_REX_ signal at 3.5 ppm will include other CEST signal sources in addition to amide protons, such as the exchangeable protons from amine, guanidinium, and hydroxyl bonds.

In summary, we applied a CEST pipeline including fast acquisition with snapshot CEST, and individual isolation of APT and NOE effect based on Lorentzian fitting and using LD and MTR_REX_ for CEST evaluation, to investigate the clinical value of CEST imaging on non-enhancing glioma. Appropriate TC and CNR to separate tumor from healthy tissue was found for LD APT and MTR_REX_ NOE with a *B*_1_ of 1.5 µT. We showed that within one CEST acquisition at 1.5 µT (< 5 min), the analysis of both LD APT and MTR_REX_ NOE allows for observing tumor heterogeneity and provided additional knowledge to conventional structural scans, which will be beneficial in future studies focused on non-enhancing glioma. It is also important to test the reproducibility of CEST imaging for robust diagnosis, e.g., comparing CEST metrics between different patients scanned on different scanners and for longitudinal observations in follow-up of individual patients, which will be included in future work to bring our current pipeline towards clinical application for glioma imaging at 3 T.
