# Correction to: 3D APT and NOE CEST-MRI of healthy volunteers and patients with non-enhancing glioma at 3 T

**DOI:** 10.1007/s10334-022-01002-w

**Published:** 2022-02-05

**Authors:** Yulun Wu, Tobias C. Wood, Fatemeh Arzanforoosh, Juan A. Hernandez-Tamames, Gareth J. Barker, Marion Smits, Esther A. H. Warnert

**Affiliations:** 1 Department of Radiology and Nuclear Medicine, Erasmus MC, Dr. Molewaterplein 40, 3015 GD Rotterdam, The Netherlands; 2 Brain Tumor Centre, Erasmus MC Cancer Institute, Rotterdam, The Netherlands; 3 Centre for Neuroimaging Science, King’s College London, London, UK

## Correction to: Magnetic Resonance Materials in Physics, Biology and Medicine 10.1007/s10334-021-00996-z

The original version of this article unfortunately contained a mistake. The following author names were incorrectly structured.

Tobias C. Wood

Gareth J. Barker

Juan A. Hernandez-Tamames

Esther A. H. Warnert

The original article has been corrected.

